# Myelodysplastic Neoplasms (MDS) with Ring Sideroblasts or *SF3B1* Mutations: The Improved Clinical Utility of World Health Organization and International Consensus Classification 2022 Definitions, a Single-Centre Retrospective Chart Review

**DOI:** 10.3390/curroncol31040134

**Published:** 2024-03-29

**Authors:** Shamim Mortuza, Benjamin Chin-Yee, Tyler E. James, Ian H. Chin-Yee, Benjamin D. Hedley, Jenny M. Ho, Lalit Saini, Alejandro Lazo-Langner, Laila Schenkel, Pratibha Bhai, Bekim Sadikovic, Jonathan Keow, Nikhil Sangle, Cyrus C. Hsia

**Affiliations:** 1 Department of Medicine, Division of Hematology, London Health Sciences Centre, London, ON N6A 5W9, Canada; shamim.mortuza@lhsc.on.ca (S.M.); benjamin.chin-yee@lhsc.on.ca (B.C.-Y.); ian.chinyee@lhsc.on.ca (I.H.C.-Y.); jenny.ho@lhsc.on.ca (J.M.H.); lalit.saini@lhsc.on.ca (L.S.); alejandro.lazolangner@lhsc.on.ca (A.L.-L.); 2 Department of Medicine, Division of Hematology, University of Ottawa, Ottawa, ON K1H 8M5, Canada; tyjames@toh.on.ca; 3 Department of Pathology and Laboratory Medicine, London Health Sciences Centre, London, ON N6A 5W9, Canada; ben.hedley@lhsc.on.ca (B.D.H.); laila.schenkel@lhsc.on.ca (L.S.); pratibha.bhai@lhsc.on.ca (P.B.); bekim.sadikovic@lhsc.on.ca (B.S.); nikhil.sangle@lhsc.on.ca (N.S.); 4 Edmonton Base Lab, Alberta Precision Laboratories, Edmonton, AB T2N 1M7, Canada; jonathan.keow@albertaprecisionlabs.ca

**Keywords:** myelodysplastic syndrome, MDS, ring sideroblasts, molecular pathology, next-generation sequencing, *SF3B1* mutation

## Abstract

Myelodysplastic neoplasms (MDS) with ring sideroblasts (RS) are diagnosed via bone marrow aspiration in the presence of either (i) ≥15% RS or (ii) 5–14% RS and an *SF3B1* mutation. In the MEDALIST trial and in an interim analysis of the COMMANDS trial, lower-risk MDS-RS patients had decreased transfusion dependency with luspatercept treatment. A total of 6817 patients with suspected hematologic malignancies underwent molecular testing using a next-generation-sequencing-based genetic assay and 395 MDS patients, seen at our centre from 1 January 2018 to 31 May 2023, were reviewed. Of these, we identified 39 evaluable patients as having lower-risk MDS with *SF3B1* mutations: there were 20 (51.3%) males and 19 (48.7%) females, with a median age of 77 years (range of 57 to 92). Nineteen (48.7%) patients had an isolated *SF3B1* mutation with a mean variant allele frequency of 35.2% +/− 8.1%, ranging from 7.4% to 46.0%. There were 29 (74.4%) patients with ≥15% RS, 6 (15.4%) with 5 to 14% RS, one (2.6%) with 1% RS, and 3 (7.7%) with no RS. Our study suggests that a quarter of patients would be missed based on the morphologic criterion of only using RS greater than 15% and supports the revised 2022 definitions of the World Health Organization (WHO) and International Consensus Classification (ICC), which shift toward molecularly defined subtypes of MDS and appropriate testing.

## 1. Introduction

Myelodysplastic neoplasms (MDS) are a heterogeneous group of clonal hematopoietic stem cell malignancies characterized by ineffective hematopoiesis. They are associated peripheral cytopenias and a risk of transformation into acute myeloid leukemia [1,2]. Patients with MDS with ring sideroblasts (MDS-RS) form a unique subtype of MDS patient and have evolving definitions [3]. The early criteria used for RS required the presence of more than 15% ring sideroblasts, where abnormal sideroblasts were defined as having five or more iron granules that encircled one third or more of the nucleus [4,5,6]. In recent years, advances in molecular testing have demonstrated that *SF3B1* (Splicing Factor 3B Subunit 1A) mutations are the most frequently observed molecular alteration in MDS patients. In 2016, the World Health Organization (WHO) classification defined ≥5% RSs in the presence of an *SF3B1* mutation as also being sufficient for a diagnosis of MDS-RS [7]. Recent studies have shown that the percentage of ring sideroblasts is not prognostically relevant [8]. Moreover, several groups have demonstrated MDS associated with *SF3B1* mutation to be a distinct MDS subtype [9,10,11]. As such, the most recent 2022 WHO Classification along with the 2022 International Consensus Classification (ICC) have moved away from relying on morphologic features and have defined a new genetic subtype: MDS with low blasts and *SF3B1* mutations (MDS-*SF3B1*) [12,13]. However, WHO 2022 does allow for the detection of ≥15% ring sideroblasts to substitute for an *SF3B1* mutation [13].

Currently, the mainstay of treatment for patients with MDS-RS includes supportive transfusions, erythropoiesis-stimulating agents (ESAs), and now also erythropoiesis maturation agents (EMAs), such as luspatercept [1,2,14]. Luspatercept is a first-in-class erythroid maturation agent that binds to selected TGF-β superfamily ligands, inhibiting aberrant Smad2/3 signaling and enhancing late-stage erythropoiesis [15]. In the MEDALIST trial and interim analysis of the COMMANDS trial, lower-risk MDS-RS patients showed decreased transfusion dependency with luspatercept [16,17]. Thus, MDS patients with less than 15% RS and *SF3B1* mutations may still benefit from luspatercept treatment. We performed a retrospective study to identify and estimate the proportion of MDS-RS patients with an *SF3B1* mutation who could be excluded based on morphologic criteria only.

## 2. Materials and Methods

### 2.1. Patient Selection

A retrospective review of all patients who underwent next-generation sequencing (NGS) testing at our tertiary centre in Southwestern Ontario, Canada, was conducted from 1 January 2018 to 31 May 2023. We included all adult patients aged 18 years or older with both a diagnosis of MDS, based on bone marrow examination, and with *SF3B1* mutation(s), confirmed via NGS testing. Records were excluded if samples were from outside our centre, or if only peripheral blood samples were available. All patients with higher-risk MDS, defined as an International Prognostic Staging System (IPSS) score ≥ 1.5 [18] and Revised IPSS (R-IPSS) score ≥ 4.0 [19], blasts greater than 5%, 5q del, or the presence of other hematologic malignancies (myeloma, lymphoma, acute or chronic leukemias, myeloproliferative neoplasms, or MDS/MPN overlap), were excluded. Data relating to patient demographics, laboratory work, diagnosis, follow-up, and management were extracted from the electronic medical records.

### 2.2. NGS Assay

All patients underwent molecular testing using the NGS-based Oncomine Myeloid Research Assay (ThermoFisher Scientific, Waltham, MA, USA), which was previously validated in our laboratory and implemented at our centre [20,21]. This assay analyzes DNA for sequence variants in 40 key target genes (17 genes with full coverage and 23 genes with partial coverage, including clinically relevant “hotspot” regions) and RNA for 29 gene fusions, including 687 fusion partners associated with hematologic disorders. Variants are classified and interpreted by genome analysts, certified clinical molecular geneticists, and/or molecular pathologists following the AMP/ASCO/CAP joint guideline [22]. All variants identified as having a variant allele frequency (VAF) ≥ 5% were assessed and classified. Variants were classified into four tiers (Tier I to IV) based on their level of clinical significance in cancer diagnosis, prognosis, and/or therapeutics [22]. Variants classified as belonging to Tiers I and II were of strong clinical significance, Tier III variants had unknown clinical significance due to the lack of significant evidence, and Tier IV variants were benign or likely to be benign. Only Tier I and II variants at VAF ≥ 5% were reported and further analyzed in this study.

### 2.3. Bone Marrow Aspirate Morphologic and RS Assessments

Bone marrow aspirate slides were prepared and stained with Wright–Giemsa solution for morphologic assessment. We used Prussian blue (Perls’ reaction) for iron staining and the determination of RS. RS were defined as five or more iron granules where the granules encircled one third or more of the nucleus. The RS percentage was enumerated based on a percentage of bone marrow erythroid precursors. A 200-cell differential was performed on bone marrow erythroid precursors where possible (required an adequate sample with at least 200 intact erythroid precursors). Enumeration was repeated on the samples by our hematopathologists [NS, JK] in a blinded fashion for confirmation.

### 2.4. Statistical Analysis

Descriptive statistics were employed to assess baseline characteristics and laboratory findings. 

## 3. Results

### 3.1. Patient Cohort

**Figure 1:**
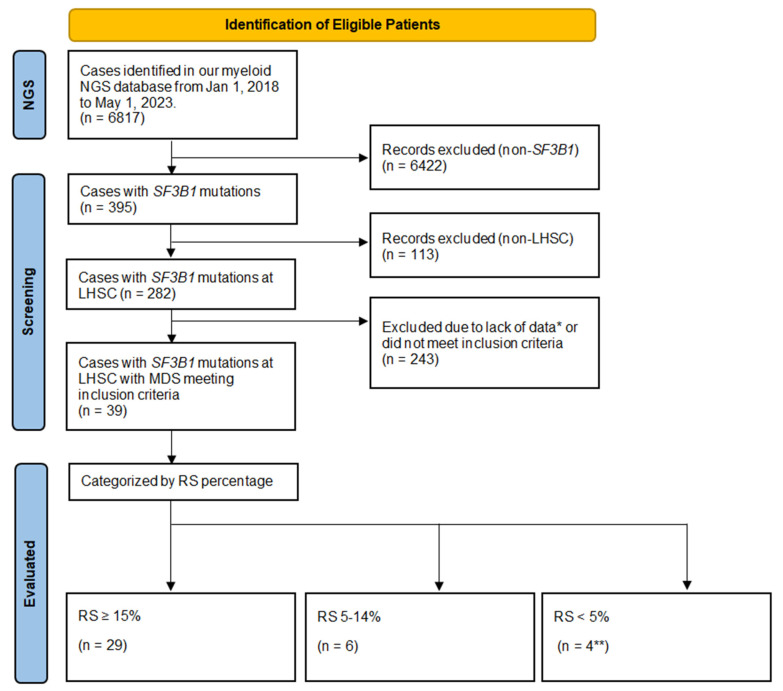
Flowchart of Patient Inclusion. Legend: NGS—next-generation sequencing; LHSC—London Health Sciences Centre; RS count—ring sideroblast count performed as a percentage of 200 hematopoietic precursors on the bone marrow; *SF3B1*—Splicing Factor 3B Subunit 1A. * Lack of clinical or laboratory data including bone marrow aspirate and biopsy performed at an outside centre prior to referral; cytogenetics—standard karyotyping, or otherwise did not meet inclusion/exclusion criteria. ** One patient had an RS count of 1% and three patients had 0%.

A total of 6817 patients underwent NGS testing from 1 January 2018 to 31 May 2023 (Figure 1). Given the retrospective nature of this study, there were no restrictions on the ordering physician in terms of which patients to test and they were able to order testing of both peripheral blood and bone marrow samples. Thus, any patient presenting with cytopenias (anemia, thrombocytopenia, neutropenia, bicytopenia, or pancytopenia), cytoses (polycythemia/erythrocytosis, thrombocytosis, neutrophilia, or panmyelosis), symptoms or signs (such as fever, night sweats, weight loss, or splenomegaly), or other indications for testing could have undergone an NGS test and it would be included in our database. A breakdown of the percentages of various conditions in terms of benign versus malignant and lymphoid versus myeloid had previously been performed in our cohort [20].

Of the 395 patients with *SF3B1* mutations, 113 were excluded as they were not patients investigated at our centre. Of the remaining 282 (395–113) patients, there were 39 that met inclusion criteria. We excluded 243 patients due to alternate diagnoses such as myeloproliferative neoplasms, other hematologic malignancy (such as myeloma or lymphoma), higher-risk MDS, or missing data (such as lack of a bone marrow aspirate/biopsy). Of the 39 evaluable patients that met the inclusion criteria, there were 20 (51.3%) males and 19 (48.7%) females, with a median age of 77 years (range 57–92). The demographics, laboratory, management, and outcome characteristics for these patients are summarized in Table 1. 

Prognostic risk scores are outlined in Table 2. The IPSS scores were low, with a score of 0 in 7 (18%) patients, and intermediate-1, with a score of 0.5 in 32 (82%) patients. The R-IPSS scores ranged from 1 to 2.5 with 10 (25.6%) and 29 (74.4%) patients categorized as “Very Low” or “Low”, respectively. Molecular IPSS (M-IPSS) scores [23] ranged from −2.69 to −0.59, with 15 (38.5%) and 24 (61.5%) patients categorized as “Very Low” or “Low”, respectively. There were 18 (46.1%) patients who received at least one red blood cell transfusion during the study period, with 15 (38.5%) being transfusion-dependent, defined as having two or more units of red blood cell transfusions during any 8 week period. Seventeen (43.6%) received an ESA and five (12.8%) received luspatercept, with three (7.7%) receiving both in a sequential manner whereby ESA treatment is followed by luspatercept. The median follow-up from the time of diagnosis until the last follow-up or death was 35 months (1–180) for this cohort.

### 3.2. Ring Sideroblasts

RS enumeration, specific *SF3B1* mutations and allele frequencies are shown in Table 3. Almost half the patients, 19 (48.7%), had an isolated *SF3B1* mutation. Additional molecular mutations were present in 20 (51.2%) patients, with TET2 (14 cases; 35.9%), and DNMT3A (8 cases; 20.5%) mutations being the most common. Further, 33 (84.6%) patients had normal karyotypes, with others having a single abnormality of del 20q, del 13q, inv 13q, or Y minus. There were 29 (74.4%) patients with ≥15% RS, 6 (15.4%) with 5 to 14% RS, one (2.6%) with 1% RS, and 3 (7.7%) with no RS. Iron stores were present in the bone marrow Prussian blue staining (Perls’ reaction) in all cases and thus would not be a limiting factor for quality of RS enumeration. During the study period, the WHO 2016 classification was utilized and 23 (59.0%) patients had MDS with RS and single-lineage dysplasia (MDS-RS-SLD), 7 (17.9%) had MDS with RS with multilineage dysplasia (MDS-RS-MLD), 7 (17.9%) had MDS-SLD, and 2 (5.1%) had MDS-MLD.

### 3.3. Patients with SF3B1 Mutation and No RSs

One patient was a 64-year-old woman with 1+ iron stores and an *SF3B1* VAF of 38.9%. She was transfusion-dependent with a serum erythropoietin (EPO) level of 615 IU/L and was treated successfully with luspatercept, becoming transfusion-independent. The second patient was an 81-year-old man with 2+ iron stores and an *SF3B1* VAF of 7.4% and was not transfusion-dependent. The remaining patient was a 79-year-old man with 3+ iron and an *SF3B1* VAF of 21.8%. He was transfusion-dependent with a serum EPO level of 59.2 IU/L and was treated with an ESA only and did not require luspatercept during the study period. 

### 3.4. Patient Outcomes 

Of the 39 patients, there were 14 deaths (35.9%). There were no patients showing disease progression or documented death due to their MDS. In 10 patients, the deaths were documented as unrelated and occurring due to falls and trauma (3), underlying cardiac issues (3), renal failure (1), Crohn’s disease (1), and other malignancies (2). For four patients, the cause of death was unknown. The median duration of follow-up from the time of diagnosis to the last follow-up assessment or death was 35 months (range 3–180) in the 39 patients overall. The median overall survival (OS) was 37.5 months (range 3–180) in the 14 patients who died.

We identified 19 (48.7%) patients with isolated *SF3B1* mutations; the median age was 76 years (57–90), with 10 females and 9 males. The median hematologic indices were WBC 7 × 10^9^/L (4–12), haemoglobin 97 g/L (65–151), MCV 105 fL (90–124), platelets 245 × 10^9^/L (24–651), and neutrophils 4 × 10^9^/L (2–10). Prognostic scores showed IPSS scores of 0 and 0.5 in 18 patients and 1 patient, respectively, R-IPSS was very low and low in 5 and 14 patients, respectively, and M-IPSS was very low and low in 12 and 7 patients, respectively. Nine patients experienced a transfusion, eight were deemed transfusion-dependent, ten patients were exposed to ESAs, and one patient received an EMA. The median duration of follow-up from the time of diagnosis to last appointment or death was 37 months (11–137). There were seven deaths, with a median overall survival of 44 months (19–137) from the time of MDS diagnosis.

There were 20 (51.3%) patients with *SF3B1* plus other molecular mutations; the median age was 79 years (64–92), with 9 females and 11 males. The median hematologic indices were WBC 6 × 10^9^/L (2–12), haemoglobin 94 g/L (71–111), MCV 105 fL (89–123), platelets 398 × 10^9^/L (46–398), and neutrophils 3 × 10^9^/L (1–9). Prognostic scores showed IPSS scores of 0 and 0.5 in 14 and 6 patients, respectively, R-IPSS was very low and low in 5 and 15 patients, respectively, and M-IPSS was very low and low in 3 and 17 patients, respectively. Nine patients experienced a transfusion, seven were deemed transfusion-dependent, seven patients were exposed to an ESA, and four patients received an EMA. The median duration of follow-up from the time of diagnosis to last appointment or death was 19 months (3–180). There were six deaths, with a median overall survival of 14 months (3–180) from the time of MDS diagnosis.

## 4. Discussion

Overall, our findings were consistent with the reported literature showing that patients with lower-risk MDS with *SF3B1* mutation(s) were older, had low or very low R-IPSS, and normal to elevated platelets [9,10,11]. We identified patients with *SF3B1* mutations with lower RS counts: there were 6 (15.4%) patients with 5 to 14% and 4 (10.3%) patients with less than 5%. This study demonstrates that the morphological presence of RS in MDS patients (as defined by ≥15% RS by morphology alone) yields a sensitivity of only 74% for the diagnosis of MDS with *SF3B1*. Instead, based on WHO & ICC 2022 guidelines, we suggest that upfront molecular testing on appropriately triaged bone marrow specimens would yield increased diagnostic accuracy for patients with MDS with *SF3B1*, who may eventually be eligible for luspatercept treatment [9,10]. We acknowledge that this molecular-first approach should be used judiciously to reduce unnecessary cytogenetic testing while increasing the chances of capturing these *SF3B1* mutations [24]. Myeloid NGS testing should not be ordered indiscriminately upfront in all patients being worked up for bone marrow disorders, but should be conducted in all patients with an established diagnosis of lower-risk MDS. This may require DNA banking samples in the initial bone marrow aspirate for subsequent testing once MDS is established, as outlined previously [24].

The classification and definition of MDS with RS has evolved greatly and, in many jurisdictions, is used to determine access to funding for various medications. In the earliest 1976 MDS classification, later revised in 1982, refractory anemia with ringed sideroblasts (RARS) was defined as a subtype of MDS with greater than 15% RS in the bone marrow [25,26] and was associated with good prognosis and responses to ESAs. Subsequently, in the WHO 2001 classification, RS could be associated with RARS with multilineage dysplasia (MLD) [4,5]. In the WHO classification of 2008, “ringed” became “ring” sideroblasts and RARS was redefined as a dysplasia that is limited to erythroid lineage and has RS ≥ 15% of bone marrow erythroid precursors. However, refractory cytopenia with MLD was defined as its own combined entity regardless of the RS count [2,26]. The 2016 WHO revision included both entities of MDS with RS with single-lineage dysplasia (SLD) or with MLD [7]. They recommended the diagnosis of MDS-RS with ≥5% (otherwise ≥15%) RS in the presence of *SF3B1* mutations. Finally, in their most recent iteration, the 2022 WHO definition as well as the new 2022 ICC definition, these bodies have moved away from relying on morphologic features and begun to place more emphasis on molecular mutations [9,10]. Nevertheless, WHO 2022 provided a caveat that the detection of ≥15% ring sideroblasts may substitute for *SF3B1* mutation and stated that an acceptable related terminology for this would be MDS with low blasts and ring sideroblasts in the absence of an *SF3B1* mutation [10].

The clinical spectrum of MDS varies from a relatively indolent course to early progression into acute myeloid leukemia and death [1,2,5,27]. The majority of patients present with cytopenias, with anemia being the most common [14]. The mainstay of treatment for lower-risk MDS patients includes transfusions, which come with all their inherent risks, challenges, and complications [1,2,14]. Various treatment options have been tried in terms of reducing the transfusion burden in these patients, including ESAs and now EMAs, such as luspatercept. A small prospective phase 3 randomized controlled trial revealed that patients receiving an ESA, with or without a granulocyte colony-stimulating factor (G-CSF) and supportive care, had improved hematologic responses. However, they had similar survival and leukemic transformations to patients receiving supportive care only [28]. Their study found that survival was increased in responders versus non-responders [28] and this was supported by another study with ESA and G-CSF in lower-risk MDS patients [29]. Various predictors of patient responses to ESA have been developed [30]. However, there remain patients who either do not respond to ESAs or relapse after treatment. In these individuals, novel therapies, including maturation agents (EMAs) such as luspatercept, have been developed to fill in the gap. Molecular testing has enabled more individualized risk stratification and management for MDS patients [31]. In particular, MDS patients with RS or *SF3B1* mutations respond to luspatercept, making it imperative to correctly identify this cohort [16,17]. In our cohort, 6 (15.4%) patients with 5 to 14% RS would potentially be missed and not qualify for luspatercept funding in our jurisdiction if molecular testing for *SF3B1* was not performed.

In our retrospective cohort, there were 14 deaths (35.9%). This high death rate for a lower-risk and good-prognosis MDS population was due to several factors. This was a small cohort with evaluable data and these patients may have been diagnosed with MDS years before NGS testing was available. The median duration of follow-up from the time of diagnosis to the last follow-up assessment or death was 35 months (range 3–180) in the 39 patients, with a median overall survival (OS) of 37.5 months (range 3–180) in the 14 patients who died. Further, the length of follow-up was potentially underestimated as some patients may have had MDS previously and then remained undetected due to the pandemic causing investigations, including NGS and bone marrow testing, to be deferred. Despite the number of deaths recorded, none of the patients saw disease progression or were documented as dying due to their MDS. Other than the 14 patient deaths, there was no disease progression or transformation into acute leukemia in the remaining 25 patients. We only included MDS, with *SF3B1* patients meeting the inclusion criteria for lower-risk MDS. This underestimated the true number of patients who were eligible at the time of diagnosis. Our NGS panel became available after 1 January 2018 and patients diagnosed with MDS prior to that date but showing disease progression at the time of NGS testing were not included in our study. This exclusion of higher-risk MDS patients from our cohort allowed us to study patients with MDS with *SF3B1* to determine which could be candidates for lower-risk treatments, such as luspatercept. Unfortunately, in this retrospective study, we lacked the data to distinguish those patients displaying a higher-risk disease at the time of NGS testing but who had lower-risk disease at the time of the initial diagnosis of MDS. Unfortunately, this also meant that we missed patients with MDS with *SF3B1* who displayed lower-risk disease at diagnosis but who since transformed. 

We compared the group of 19 (48.7%) patients with only *SF3B1* mutations to the group of 20 (51.3%) patients with *SF3B1* plus other molecular mutations. Both groups were similar in age, sex distribution, hematologic indices, IPSS, and R-IPSS scores. They differed in their M-IPSS scores, as expected, with the additional mutations in the latter group. Although both groups had similar percentages of exposure to transfusions and transfusion dependency, the latter group seemed to have a shorter overall survival. Median overall survival was 44 months in the seven patients with only *SF3B1* mutations, compared to 14 months in the six patients with *SF3B1* plus other mutations who died. However, this was not statistically significant (*p* = 0.37).

There are several limitations to our study, as already noted above. As a single-centre, retrospective study, we were not able to include data on patients with MDS who were co-managed at our centre and whose diagnostic testing was performed elsewhere. There may have been a small number of patients with a diagnosis of MDS who did not undergo upfront NGS testing, although it was routinely performed in our patients with MDS during the study period. In order to focus on the subset of patients with lower-risk MDS patients with *SF3B1* mutations, we excluded any patients with elevated blasts, 5q del, or other malignancies. We appreciate that there may be some patients with lower-risk MDS, blasts of 5–9%, with co-existing 5q del, or other malignancies that may not preclude the use of ESAs or luspatercept. Further, our final 39 MDS patients with *SF3B1* meeting the inclusion criteria still represented an underestimate of the true number of patients who would have been eligible at the time of diagnosis. Thus, we caution against extrapolating our outcome data to all MDS patients. However, the goal of this study was not to study all the outcomes of various subgroups of MDS, including those with *SF3B1* mutations, but to demonstrate that there are patients being excluded from the updated WHO classification based on morphologic assessments alone.

## 5. Conclusions

Overall, our findings support the revised 2022 WHO and ICC classifications, which shift toward molecularly defined subtypes of MDS. Although we do not encourage the indiscriminate use of molecular testing for undiagnosed patients with cytopenias, we do suggest molecular testing for patients with an established diagnosis of lower-risk MDS, with or without ring sideroblasts.

## Figures and Tables

**Table 1:** Baseline demographics and characteristics.

	Total Patients	Male	Female
**Patient Characteristics**			
Total Patients; number (%)	39 (100)	20 (51)	19 (49)
Age; median (range) years	77 (57–92)	78 (57–90)	76 (64–92)
Laboratory Findings *			
Haemoglobin; median (range) g/L	96 (65–151)	95 (65–151)	96 (71–112)
MCV; median (range) fL	105 (89–124)	108 (89–124)	104 (93–117)
Leukocytes; median (range) × 10^9^/L	6 (2–12)	6 (2–12)	6 (4–12)
Platelets; median (range) × 10^9^/L	220 (24–651)	203 (24–651)	253 (54–371)
Ferritin; median (range) mcg/L	554 (59–4519)	562 (59–4519)	544 (172–2894)
Serum EPO; median (range) IU/L	43 (9–1070)	34 (9–1070)	47 (18–615)
**Management and Outcomes**			
Transfusions **; number (%)	18 (46)	8 (40)	10 (53)
Transfusion-dependent ***; number (%)	15 (38)	7 (35)	8 (42)
ESA; number (%)	17 (44)	8 (40)	9 (47)
EMA; number (%)	5 (13)	1 (5)	4 (21)
Other treatments; number (%)	2 (5)	2 (10)	0
Duration of follow-up †; median (range) months	35 (3–180)	34 (3–137)	37 (4–180)

Legend: MCV—mean cell volume; EPO—erythropoietin; ESA—erythropoiesis-stimulating agent; EMA—erythropoiesis maturation agent. * Laboratory findings at time of myelodysplastic syndrome diagnosis. ** Transfusions—patient experienced any red cell transfusion during the study period. *** Transfusion-dependent—patient was exposed to greater than 2 units of red blood cells during any 8-week period during the study period from 1 January 2018 until 31 May 2023. † Duration of follow-up-from time of diagnosis to last follow-up or death.

**Table 2:** Characteristics of underlying MDS.

	Total Patients *N* = 39	Male *N* = 20	Female *N* = 19
IPSS Risk Classification			
Low; *n* (%)	32 (82%)	17 (85%)	15 (79%)
Intermediate-1; *n* (%)	7 (18%)	3 (15%)	4 (21%)
R-IPSS Risk Classification			
Very Low; *n* (%)	10 (26%)	6 (30%)	4 (21%)
Low; *n* (%)	29 (74%)	14 (70%)	15 (79%)
M-IPSS Classification			
Very Low, *n* (%); Score median (range)	15 (39%); −1.64 (−2.69 to −1.52)	7 (35%); −1.66 (−2.69 to −0.85)	8 (42%); −1.63 (−1.93 to −1.57)
Low, *n* (%); Score median (range)	24 (62%); −1.13 (−1.46 to −0.59)	13 (65%); −1.17 (−1.46 to −0.59)	11 (58%); −1.09 (−1.46 to −0.81)

Legend: IPSS—International Prognostic Scoring System; R-IPSS—Revised International Prognostic Scoring System; M-IPSS—Molecular International Prognostic Scoring System.

**Table 3:** Patient level data.

Patient Serial ID	Age (yrs)	Sex	MDS Subtype (WHO 2016)	RS Count (%)	*SF3B1* Mutation VAF (%)	Other Gene Mutations	Cytogenetics
1	76	F	RS-SLD	15	*SF3B1*:c.2098A>G,p.(Lys700Glu) (23.4%)	None	Normal
2	78	M	RS-SLD	15+	*SF3B1*:c.1997A>C,p.(Lys666Thr) (38%)	None	Normal
3	76	F	RS-SLD	15+	*SF3B1*:c.2098A>G,p.(Lys700Glu) (42.2%)	*TET2*	Del 13q
4	64	F	SLD	0	*SF3B1*:c.2098A>G,p.(Lys700Glu) (38.9%)	*TET2*, *TP53*	Normal
5	64	F	SLD	9	*SF3B1*:c.2098A>G,p.(Lys700Glu) (40.8%)	None	Normal
6	82	F	MLD	10	*SF3B1*:c.1873C>T,p.(Arg625Cys) (33.8%)	None	Normal
7	83	M	RS-MLD	15+	*SF3B1*:c.2098A>G,p.(Lys700Glu) (39.8%)	*DNMT3A*	Normal
8	65	F	RS-SLD	15+	*SF3B1*:c.2098A>G,p.(Lys700Glu) (45%)	*IDH2*, *DNMT3A*	Normal
9	84	F	RS-MLD	15+	*SF3B1*:c.2098A>G,p.(Lys700Glu) (40%)	*TET2*, *DNMT3A*	Normal
10	64	M	RS-SLD	15+	*SF3B1*:c.2098A>G,p.(Lys700Glu) (25.6%)	None	Normal
11	88	F	SLD	5–14%	*SF3B1*:c.2098A>G,p.(Lys700Glu) (43.2%)	*TET2*, *DNMT3A*, *SH2B3*	Del 20q
12	73	M	RS-SLD	15+	*SF3B1*:c.2098A>G,p.(Lys700Glu) (35.9%)	*TET2*	Inv 13q
13	74	M	RS-MLD	15+	*SF3B1*:c.2098A>G,p.(Lys700Glu) (36.1%)	*KIT*	Normal
14	80	F	RS-SLD	15+	*SF3B1*:c.1873C>T,p.(Arg625Cys) (26.8%)	None	Normal
15	85	M	RS-SLD	15+	*SF3B1*:c.1997A>G,p.(Lys666Arg) (36.6%)	*TET2*	Normal
16	65	F	RS-SLD	15+	*SF3B1*:c.2098A>G,p.(Lys700Glu) (28.5%)	None	Normal
17	63	M	RS-SLD	15+	*SF3B1*:c.1986C>G,p.(His662Gln) (29.5%)	None	Y minus
18	90	M	MLD	8	*SF3B1*:c.2098A>G,p.(Lys700Glu) (39.2%)	None	Y minus
19	78	M	RS-SLD	15+	*SF3B1*:c.1997A>G,p.(Lys666Arg) (33.3%)	None	Normal
20	67	M	RS-SLD	15+	*SF3B1*:c.2098A>G,p.(Lys700Glu) (44.6%)	None	Normal
21	57	M	SLD	12	*SF3B1*:c.1998G>T,p.(Lys666Asn) (33.5%)	None	Normal
22	80	F	RS-MLD	15+	*SF3B1*:c.2098A>G,p.(Lys700Glu) (37.9%)	*TET2*	Normal
23	81	M	MLD	0	*SF3B1*:c.1986C>G,p.(His662Gln) (7.4%)	*TET2*, *KIT*	Normal
24	70	F	RS-SLD	15+	*SF3B1*:c.1984C>T,p.(His662Tyr) (45.6%)	*TET2*	Normal
25	92	F	RS-SLD	15+	*SF3B1*:c.2098A>G,p.(Lys700Glu) (42.7%)	*TET2*	Normal
26	79	M	SLD	0	*SF3B1*:c.1998G>C,p.(Lys666Asn) (21.8%) *SF3B1*:c.2098A>G, p.(Lys700Glu) (20.1%)	None	Normal
27	86	M	RS-SLD	15+	*SF3B1*:c.2098A>G,p.(Lys700Glu) (28.7%)	*DNMT3A*	Normal
28	88	M	SLD	1	*SF3B1*:c.1997A>C,p.(Lys666Thr) (39.6%)	None	Normal
29	70	F	RS-SLD	15+	*SF3B1*:c.1986C>A,p.(His662Gln) (37.4%)	None	Normal
30	81	M	RS-SLD	65	*SF3B1*:c.2098A>G,p.(Lys700Glu) (46%)	*DNMT3A*, *TET2*	Normal
31	76	F	RS-SLD	15+	*SF3B1*:c.2098A>G,p.(Lys700Glu) (38.5%)	None	Normal
32	78	F	RS-SLD	15+	*SF3B1*:c.2098A>G,p.(Lys700Glu) (40.8%)	None	Normal
33	87	F	SLD	5–14%	*SF3B1*:c.2098A>G,p.(Lys700Glu) (32.2%)	None	Normal
34	75	M	RS-SLD	15+	*SF3B1*:c.1997A>G,p.(Lys666Arg) (43.6%)	*ZRSR2*, *TET2*	Normal
35	73	M	RS-SLD	15+	*SF3B1*:c.2098A>G,p.(Lys700Glu) (31.7%)	*DNMT3A*	Normal
36	73	F	RS-SLD	15+	*SF3B1*:c.2098A>G,p.(Lys700Glu) (37.1%)	None	Del 20q
37	81	M	RS-SLD	15+	*SF3B1*:c.2098A>G,p.(Lys700Glu) (25.1%)	*DNMT3A*	Normal
38	77	F	RS-MLD	15+	*SF3B1*:c.1986C>A,p.(His662Gln) (23.4%)	*TET2*	Normal
39	75	M	RS-MLD	15+	*SF3B1*:c.2098A>G,p.(Lys700Glu) (39.7%)	*TET2*	Normal

Legend: WHO—World Health Organization; RS Count—ring sideroblast count performed as a percentage of 200 hematopoietic precursors on the bone marrow; *SF3B1*—Splicing Factor 3B Subunit 1A; VAF—variant allele frequency; other molecular and cytogenetics were performed on the bone marrow samples; normal cytogenetic karyotype—46,XX for females and 46,XY for males.

## Data Availability

All data generated or analyzed during this study are available upon request to the corresponding author.
